# Teosinte in Europe – Searching for the Origin of a Novel Weed

**DOI:** 10.1038/s41598-017-01478-w

**Published:** 2017-05-08

**Authors:** Miluse Trtikova, Andre Lohn, Rosa Binimelis, Ignacio Chapela, Bernadette Oehen, Niklaus Zemp, Alex Widmer, Angelika Hilbeck

**Affiliations:** 1 ETH Zurich, Institute of Integrative Biology (IBZ), Universitätstrasse 16, 8092 Zurich, Switzerland; 2 Agroecology and Food Systems Chair, Universitat de Vic – Universitat Central de Catalunya, c/de la Laura 13, 08500 Vic, Spain; 3 University of California Berkeley, Department of Environmental Science, Policy and Management, 108 Hilgard Hall, 94720 Berkeley, USA; 4 ETH Zurich, Genetic Diversity Centre (GDC), Universitätstrasse 16, 8092 Zurich, Switzerland

## Abstract

A novel weed has recently emerged, causing serious agronomic damage in one of the most important maize-growing regions of Western Europe, the Northern Provinces of Spain. The weed has morphological similarities to a wild relative of maize and has generally been referred to as teosinte. However, the identity, origin or genetic composition of ‘Spanish teosinte’ was unknown. Here, we present a genome-wide analysis of single-nucleotide polymorphism (SNP) data for Spanish teosinte, sympatric populations of cultivated maize and samples of reference teosinte taxa. Our data are complemented with previously published SNP datasets of cultivated maize and two Mexican teosinte subspecies. Our analyses reveal that Spanish teosinte does not group with any of the currently recognized teosinte taxa. Based on Bayesian clustering analysis and hybridization simulations, we infer that Spanish teosinte is of admixed origin, most likely involving *Zea mays* ssp. *mexicana* as one parental taxon, and an unidentified cultivated maize variety as the other. Analyses of plants grown from seeds collected in Spanish maize fields and experimental crosses under controlled conditions reveal that hybridization does occur between Spanish teosinte and cultivated maize in Spain, and that current hybridization is asymmetric, favouring the introgression of Spanish teosinte into cultivated maize, rather than vice versa.

## Introduction

Increasing human transfer of plants across geographical regions leads to frequent introductions of non-native plant species around the world. If these introduced taxa meet close relatives, hybridization and introgression can occur^1^. The inadvertent introduction of species closely related to major crop plants may lead to hybridization and the formation of weedy lineages^2^. Alternatively, weedy taxa can also evolve directly from a domesticated ancestor^3^, as in the case of some weedy rice populations^4, 5^. Weedy lineages can incur massive yield losses and cause major costs^6^.

Maize is the third most important crop plant in Spain with production reaching almost 4.7 million tonnes^7^. In 2009, farmers in Northern Spain (Aragon) began to observe plants in their maize fields that resembled cultivated maize before the onset of flowering but then developed highly branching phenotypes with small cobs and shattering seeds^8^. These traits are typical for teosinte, wild relatives of cultivated maize^9^. Until 2014, this so-called ‘Spanish teosinte’ has spread in Aragon and has also been reported from a neighbouring region in Catalonia^10, 11^. About 750 ha of maize cultivation have been affected so far, mostly in Aragon^11^. Due to maize monocropping, density of Spanish teosinte can become high on affected fields and may cause severe maize yield losses and high economic costs^12^. In some regions of Aragon, this weed has become the prime agronomic problem for maize farmers^10^.

Maize (*Zea mays mays*) was domesticated from its wild relative teosinte about 9,000 years ago in southern Mexico^13^. Domesticated maize, including high-yielding hybrid maize varieties grown in Europe, is strictly monopodic, with non-shattering kernels that remain tightly attached to the cob, in contrast to the shattering kernels of the cob-less, and highly branched, teosinte. Although maize cobs, or parts of them, can remain in the field and do germinate and grow feral, it is considered unlikely that they successfully establish feral populations beyond arable fields and without human support^9^. Hence, cultivated maize is generally considered to have little to no risk of causing concerns as a volunteer or feral weed^14^.

Teosinte is the common name of a group of wild grasses (Poaceae) and includes highly variable species and subspecies that occur in scattered populations in many areas across Mexico and Central America (Mesoamerica)^15^. In many areas of Mesoamerica, teosinte populations have come under serious threat with the expansion of ranching and farming and are facing a massive decline in abundance to the point of extinction of some species^16^, which forced the Mexican government to install conservation measures for their protection^17^. Thus, in their centre of origin, many teosinte populations are endangered and require protection measures, although occasionally they can also act as local weeds^18^.

Teosinte and maize belong to the same genus, *Zea*, which consists of five species: 1) perennial diploid (2n = 20) *Z. diploperennis*, 2) perennial tetraploid (2n = 40) *Z. perennis*, 3) annual diploid (2n = 20) *Z. luxurians*, 4) annual diploid (2n = 20) *Z. nicaraguensis*, and 5) the annual species *Zea mays*. The latter encompasses four annual diploid (2n = 20) subspecies: (i) ssp. *mays*, the domesticated maize, (ii) ssp. *mexicana*, (iii) ssp. *parviglumis*, and (iv) ssp. *huehuetenangensis* ^19^. *Z.m*. ssp. *mexicana* and *Z.m*. ssp. *parviglumis* are most closely related to domesticated maize, the latter subspecies being called ‘Balsas’ teosinte and considered the ancestor of cultivated maize^9^. All teosintes are believed to be endemic to Mesoamerica^20^ where cultivated maize and teosintes often grow in geographic proximity and flower synchronously. Overall, *Z.m*. ssp. *mexicana* grows in cooler, drier central highlands, mostly above 1800 m, while *Z.m*. ssp. *parviglumis* grows in warmer, wetter lower elevations in the river valleys of southern and western Mexico, mostly below 1800 m^15^.

Although it is known that all teosintes can hybridize with maize, this typically occurs at low rates even when teosinte is abundant^21^. Hybridization appears to be most common between domesticated maize and *Z.m*. ssp. *parviglumis* ^15, 22^ but gene flow does not occur reciprocally, which may explain why teosintes continue to coexist even when growing in close vicinity to much larger maize populations^21^. When teosinte pollen is applied to maize silks, resulting hybrids are vigorous and highly fertile^23^. However, when teosinte is pollinated by maize pollen, plants of *Z.m*. ssp. *mexicana* set seed very inconsistently or not at all^22^. Similarly, Hufford *et al*. found evidence of adaptive introgression of *Z.m*. ssp. *mexicana* alleles into maize during its expansion to the highlands of central Mexico, but observed very little evidence for adaptive introgression in the other direction, from cultivated maize into *Z.m*. ssp. *mexicana* ^24^.

This asymmetrical pollination success is under the control of a gene called the *‘Teosinte crossing barrier*’ (*Tcb*)^23^ which may reproductively isolate at least some teosinte species from maize. Aylor *et al*. postulated that gene flow and subsequent introgression of maize alleles into teosinte populations most likely occur when teosinte first pollinates maize^20^. The resultant hybrids would then backcross with teosinte which could lead to the introgression of maize alleles into the teosinte background. They speculated that the pollination from teosinte to maize most likely represents the rate-limiting step in the introgression of maize alleles into teosinte^20^. However, this has not been investigated yet to any conclusive extent, even though teosinte and maize have intensively been studied from a population genetics perspective, including studies utilizing DNA samples recovered from archaeological specimens^13, 24–27^.

So far, the Spanish authorities have speculated that the introduced teosinte is *Z.m*. ssp. *mexicana* ^11^. Spanish teosinte can produce long-lasting seed banks, and its control either by mechanical means, crop rotation or herbicide treatment has proven difficult^10, 11^. Knowing the origin of the novel weed in Spain may help to prevent introductions of further seed material, to monitor the spread of this weed, and to take targeted control measures in the future. The taxonomic identity of the Spanish teosinte, its introduction history, as well as its ecology and potential risks for local and neighbouring European farming systems remain largely unknown. To develop effective measures for monitoring, control and prevention, data on these aspects are fundamental. Here, we addressed the following research questions: (i) to what taxon can teosinte-like plants collected in Spain be assigned?, (ii) what is the potential origin of these Spanish teosinte lineages?, (iii) is there evidence for on-going hybridization between Spanish teosinte and commercially cultivated maize in Spain? To answer these questions, we collected Spanish teosinte and hybrid-like seeds (autumn 2014 and 2015) and leaf samples of Spanish teosinte and cultivated maize (summer 2015) in the region of Aragon, Spain, and genotyped these together with teosinte reference plants using the MaizeSNP50 BeadChip, a widely used resource for high-density genotyping of maize and its wild relatives^28–30^.

## Results

**Figure 1:**
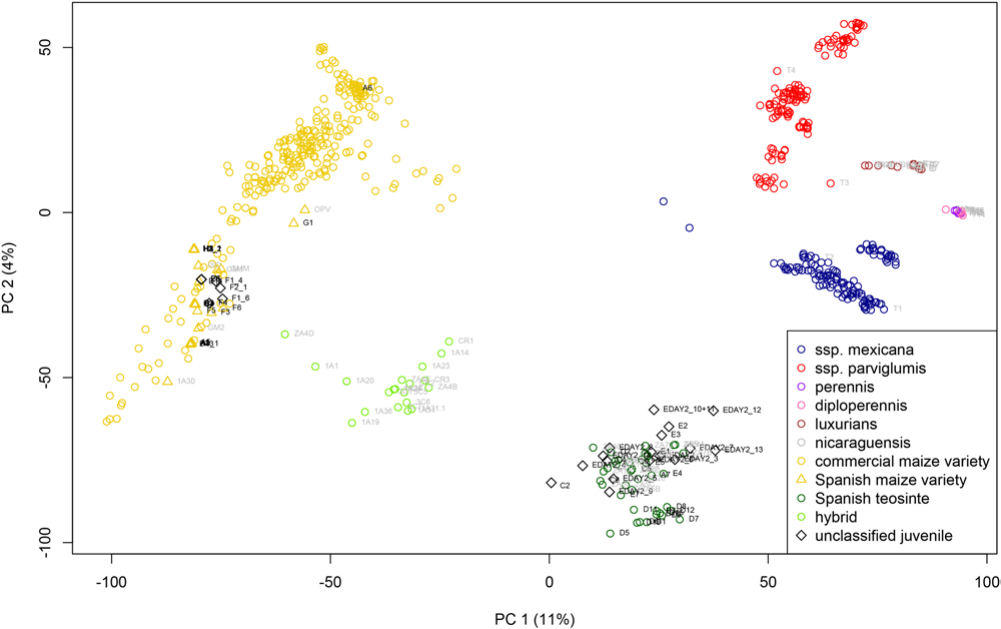
PCA of maize, teosinte and hybrid samples collected in Spain. Samples were collected from plants growing in the field (black labels), from seeds collected in the field and subsequently grown in a climate chamber (grey labels) or from reference material (grey labels). Labelled symbols represent own data that were compared to other publically available data (without labels) (27′476 SNPs and 662 individuals).

**Figure 2:**
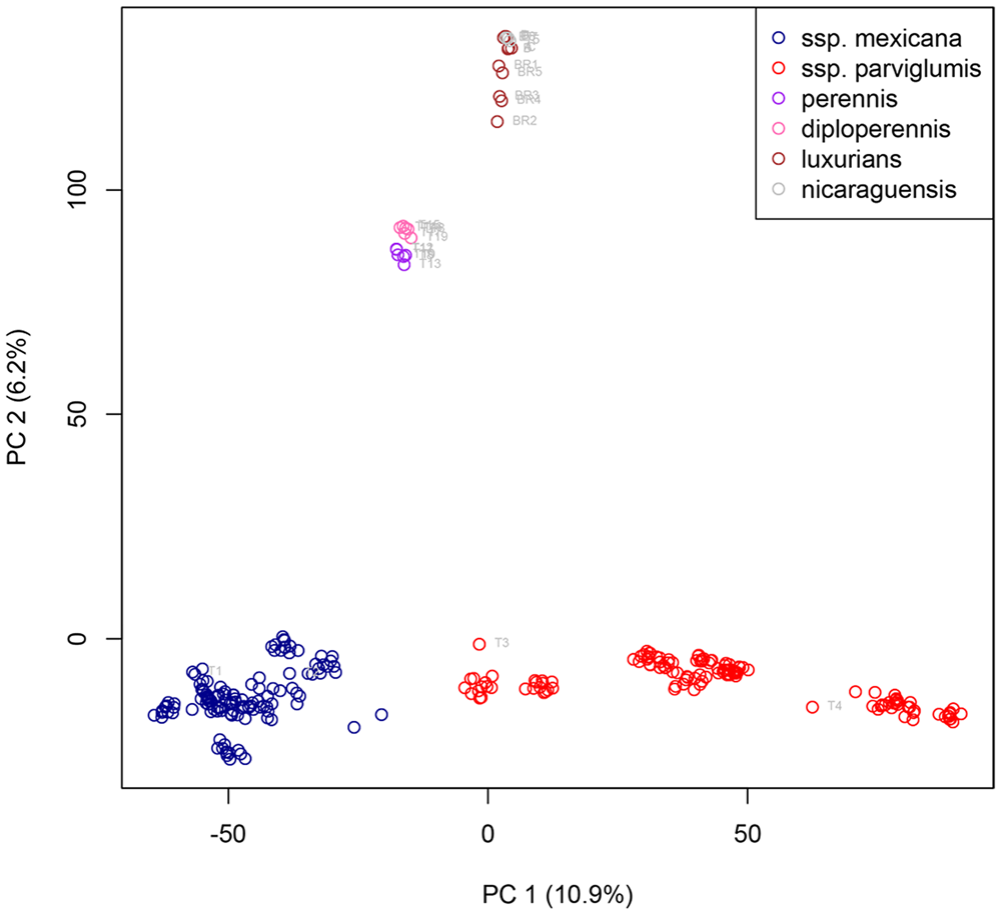
PCA of teosinte samples grown from the seeds obtained as a reference material. Labelled symbols represent own data that were compared to other publically available data (without labels) (27′476 SNPs and 280 individuals).

Using PCA, the SNP data allowed a clear separation between commercial maize varieties and all teosinte taxa included in this study (Fig. 1). Samples from Spanish maize varieties collected in the field or grown from seeds grouped with other commercial maize varieties previously analysed by Olukolu *et al*.^29^. Similarly, reference samples of *Z.m*. ssp. *mexicana* and ssp. *parviglumis* obtained from USDA grouped with samples of these species previously investigated by Pyhäjärvi *et al*.^28^ (Fig. 2). All other teosinte species obtained from CIMMYT and USDA formed separate groups (Figs 1 and 2). Teosinte from Brazil grouped with reference samples of *Z. luxurians*, confirming the results of Silva *et al*.^31^.

Samples collected in maize fields in Spain that did not represent commercial maize varieties formed two groups. One group, containing all individuals initially scored as Spanish teosinte plants, was clearly separated from commercial maize varieties but also from all currently recognized Mexican and Nicaraguan teosintes (Fig. 1). The second group of plants from Spain were intermediate between Spanish teosinte plants and commercial maize varieties (Fig. 1). Plants in this group were grown from hybrid-like seeds collected in the field that shared phenotypic features of maize and Spanish teosinte (represented by light green circles in Fig. 1). In addition, this group also encompassed our own experimental F1 hybrids between Spanish commercial maize and Spanish teosinte plants in which the teosinte served as pollen donors.

**Figure 3:**
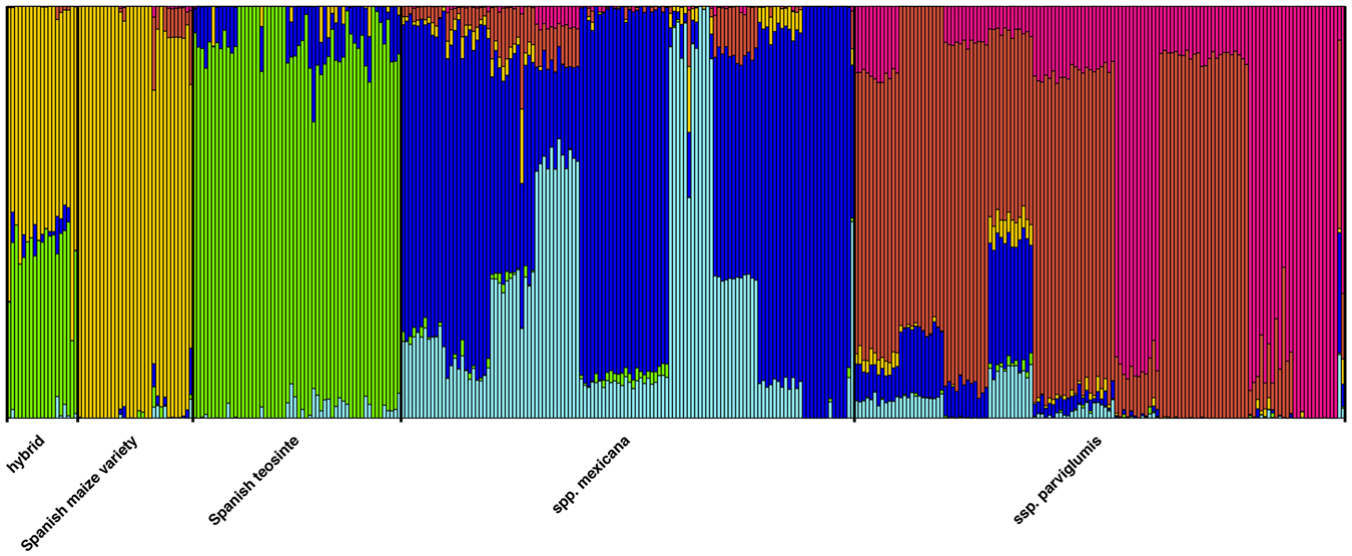
STRUCTURE analysis of Spanish maize varieties, hybrids, Spanish teosinte, *Z.m*. ssp. *mexicana* and ssp. *parviglumis*. Bar plot of assignment proportions at K = 6 combining own SNP data and other publically available data (27′476 SNPs and 360 individuals).

STRUCTURE analysis of samples from Spain, together with *Z.m*. ssp. *mexicana* and ssp. *parviglumis*, also clearly separated Spanish teosinte from the two subspecies of *Z. mays* and further revealed that Spanish teosinte shares some alleles with *Z.m*. ssp. *mexicana*. Further, individuals grown from the seeds that were collected in the field in Spain and that were intermediate between Spanish teosinte plants and Spanish commercial maize (represented by light green circles in Fig. 1) were found to be early generation hybrids between maize cultivated in Spain and Spanish teosinte (Fig. 3).

**Figure 4:**
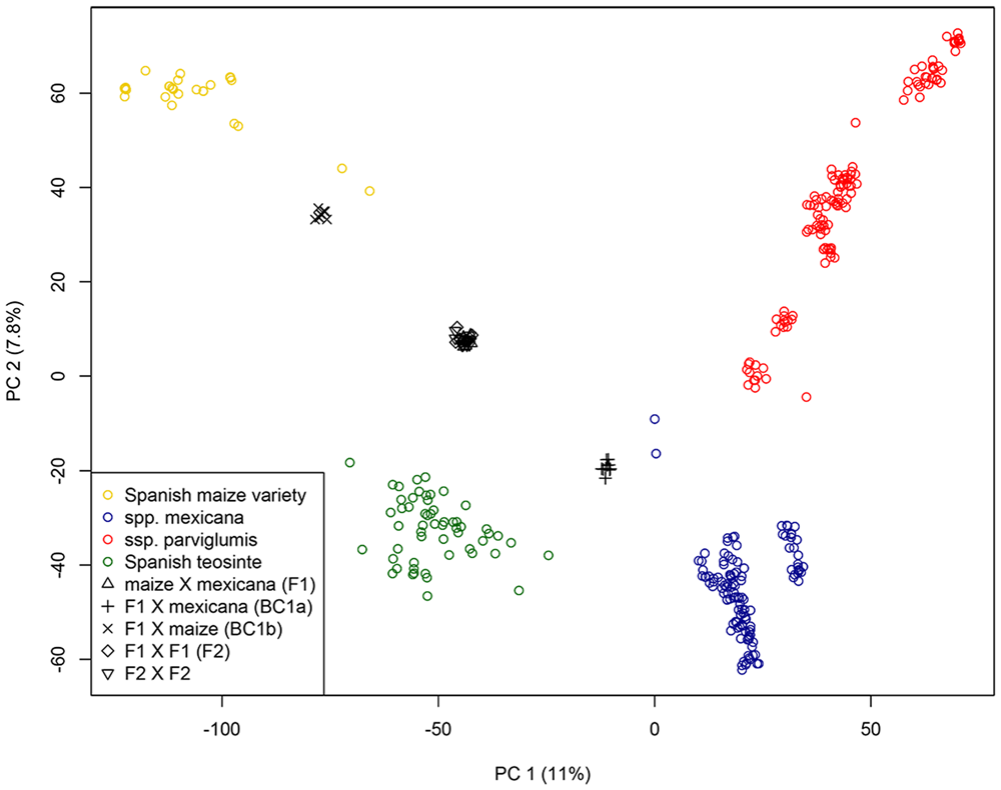
PCA of simulated hybridization between Spanish maize varieties and *Z.m*. ssp. *mexicana*. Coloured symbols represent empirical data (27′476 SNPs and 360 individuals) and black symbols represent simulated data.

Because Spanish teosinte plants did not group with any of the known Mexican and Nicaraguan teosinte taxa, we evaluated a possible admixed origin by simulating hybridization between Spanish commercial maize genotypes and different teosinte species. Results suggest that commercial maize varieties currently planted in Spain were not involved in the formation of Spanish teosinte (Fig. 4), which is also consistent with the outcome of the STRUCTURE analysis (Fig. 3).

## Discussion

The novel weed found in maize fields in Spain has been tentatively identified as a wild relative of maize, teosinte, but no systematic effort to establish its identity or ancestry has been undertaken until now. Knowing the identity of this weed is not only useful for monitoring and control, but fundamental to trace its origin and to assess its potential for interbreeding and future evolution towards a possibly even more damaging weed. We, therefore, genotyped the Spanish teosinte plants together with commercially grown maize and all currently recognized teosinte taxa from Mexico and Nicaragua. Surprisingly, the Spanish teosinte was not only clearly separated from cultivated maize but also from all teosinte species tested (Fig. 1). These results are not compatible with the published suggestion that teosinte plants observed in Spain represent *Z.m*. ssp. *mexicana* ^11^.

In one attempt at testing the hypothesis that there could be some relation between Spanish teosinte and expatriated populations of teosintes *sensu stricto*, we included material from a Brazilian population of teosinte that was introduced there as a forage species. Recently, it was reported that this teosinte from southern Brazil belonged in the species *Z. luxurians* ^31^, based on morphological traits and the location of chromosomal knobs. However, no genetic analysis had been performed to date. Our results confirmed that this Brazilian teosinte is *Z. luxurians*, as suggested by the study of Silva *et al*.^31^, but it did not associate with Spanish teosinte.

The fact that Spanish teosinte did not group with any currently recognized teosinte taxa from Mexico and Nicaragua, or with known populations of expatriated teosintes, led us to hypothesize that it might be of admixed origin. Indeed, our STRUCTURE analysis revealed that Spanish teosinte shares alleles with *Z.m*. ssp. *mexicana*. Very likely, Spanish teosinte could originate from hybridization and subsequent backcrossing between cultivated maize and *Z.m*. ssp. *mexicana*. Such a process would seem necessary to explain our data, given the current state of knowledge on the asymmetric crossing behaviour between maize and teosintes in general^20–23^. Simulated hybridization suggests that the hybridization leading to Spanish teosinte likely did not occur in very recent years. Whether such a weed had originally formed in Europe or elsewhere is open to further research.

The key hybridization event(s) leading to the emergence of Spanish teosinte could have taken place inadvertently in any of the Mesoamerican regions where native *Z.m*. ssp. *mexicana* and domesticated maize coexist, or indeed through human intervention elsewhere, including Europe, as *Z.m*. ssp. *mexicana* has been used in various breeding programs in order to improve agronomic traits of cultivated maize^32^ or to evaluate the suitability of the hybrids as a forage grass^33, 34^. Against such a single-introduction hypothesis, the origin of Spanish teosinte could also be complex, resembling the case of weedy rice in the USA, where some populations seem to have arisen via hybridization between cultivated rice and weedy rice^35^, whereas other seem to have evolved directly from cultivated rice^5, 36^.

The risk of further hybridization events between commercial maize and Spanish teosinte is worth considering in light of the possibility of further development of even more invasive weed than the population currently found in Northern Spain. The plants grown from hybrid-like seeds, which we collected in the field in Spain, proved indeed to be intermediate between cultivated maize and teosinte, having elongated lateral branches and producing bunches of ears, often combined with tassels. These samples grouped together (light green circles in Fig. 1) with our experimental F1 hybrids derived from crosses in which Spanish commercial maize plants acted as pollen recipients and Spanish teosinte as pollen donors. Thus, we suggest that further hybridization is not only possible, but is already happening in the field, albeit so far we have only encountered teosinte-to-maize hybrid seed, and not maize-to-teosinte seed.

Surprisingly, we have not detected any hybrids among the plants that we sampled directly in the field. A plausible explanation is that when maize is pollinated by teosinte, the seeds do not easily shatter, with many of them being harvested together with the commercially grown maize. Pollination occurring in the other direction, from cultivated maize to teosinte, is known to be significantly less viable, due to the control of *Teosinte crossing barrier* (*Tcb*)^23^. Lu *et al*. observed that in silk carrying the *Tcb*, pollen tubes had clustered callose plugs and their growth was slower in comparison to pollen tubes of compatible crosses^37^. Such crossing incompatibility may also exist between commercial maize and Spanish teosinte. From our exploratory crossing experiments, we can confirm that hand-pollination of Spanish teosinte with maize pollen results only rarely in viable seeds (unpublished results). Thus, while the crossing barrier may reduce the frequency with which such hybrids are formed in the field, it is important to note that it does not preclude their formation, even though at low rates. Therefore, we expect that more intensive sampling, over a longer period of time, may likely reveal the existence of maize-to-teosinte introgressants, especially if on-going hybridization between teosinte and maize leads to the formation of hybrids that are more compatible with a teosinte mother plant, as postulated by Aylor *et al*.^20^.

Further studies are needed to fully understand the evolutionary origin and demographic processes involved in the formation of Spanish teosinte. In particular, much more intensive sampling, both spatially and temporally, could reveal the amount of genetic and phenotypic diversity, as well as the extent to which the population of Spanish teosinte is changing, including through hybridization with cultivated maize. However, we now have a basis of understanding that enables necessary practical measures to confront the challenge of this serious, possibly invasive, weed. With this understanding, it should be possible to establish monitoring and mapping efforts to track, and hopefully contain, the spread of the weed. Whether this weed remains a serious problem only in the Spanish region where it is now confined, or whether it expands to other maize-growing areas in Europe and beyond may be determined by the degree with which these questions are pursued.

## Methods

### Plant material

Teosinte and hybrid-like seeds were collected from two different sites in the region of Zaragoza, Spain in autumn 2014 and 2015. In summer 2015 we surveyed the maize producing region around Zaragoza and collected leaf samples from three fallow and five standing maize fields. We sampled 3–20 plants per field (Supplementary Table S1). The leaf samples were dried and stored on silica gel for further molecular analysis.

Seeds collected in the field were germinated and grown under controlled conditions (20–25 °C, 50–65% rh, 16/8 h L/D) in climate chambers at ETH Zurich. Once matured, leaf samples were collected from 34 plants (Supplementary Table S1), dried and stored on silica gel for further molecular analysis.

Reference material of the different teosinte taxa was obtained from CIMMYT and USDA (Supplementary Table S1). Seeds of teosinte grown in Brazil were obtained from local markets in the state of Santa Catarina, Brazil. All teosinte seeds were shelled and germinated on filter paper under controlled conditions in the climate chamber (20–25 °C, 50–65% rh, 16/8 h L/D). Also, the seeds of the following maize varieties grown in Spain were germinated on filter paper: LG30490YG, ES TORQUAZ, ROJO, PR33D48, DKC66-66. The first leaves were sampled, lyophilized and stored in silica gel for further molecular analysis.

Experimental crosses were performed with six Spanish insect resistant Bt maize (LG30490YG) and 14 teosinte plants grown from the seeds collected in Spain under controlled conditions in a climate chamber (20–25 °C, 50–65% rh, 12/12 h L/D). The adventitious roots were sampled, lyophilized and stored on silica gel for further molecular analysis. Three seeds from one cob from a maize plant (mother) pollinated by teosinte were germinated and sampled in the same way as the reference material.

### SNP genotyping

DNA was extracted from dried leaf and root material with CTAB buffer, following a slightly modified protocol described in Doyle and Doyle^38^, and quantified using NanoDrop (Thermo Science). For the experimental crosses, DNA for the mother plant (Bt maize) was pooled from 6 different plant samples using the same concentration levels, similarly, for the father plant (teosinte) DNA from 14 different plant samples was pooled together. Genotyping was conducted at the UC Davis Genome Center using the MaizeSNP50 BeadChip and Infinium HD Assay (Illumina, San Diego, CA, USA).

### Data analysis

SNPs were called using GenomeStudio V2009.1 (Illumina). Gen Train score had to be larger than 0.7 to retain a SNP leading to 41,784 SNPs. SNP data were deposited in the Dryad repository (http://dx.doi.org/10.5061/dryad.60210). To the dataset we added published and publically available data on *Z.m*. ssp. *mexicana and* ssp. *parviglumis* individuals^28^ and commercial maize varieties^29^ (Supplementary Table S1).

**Figure 5:**
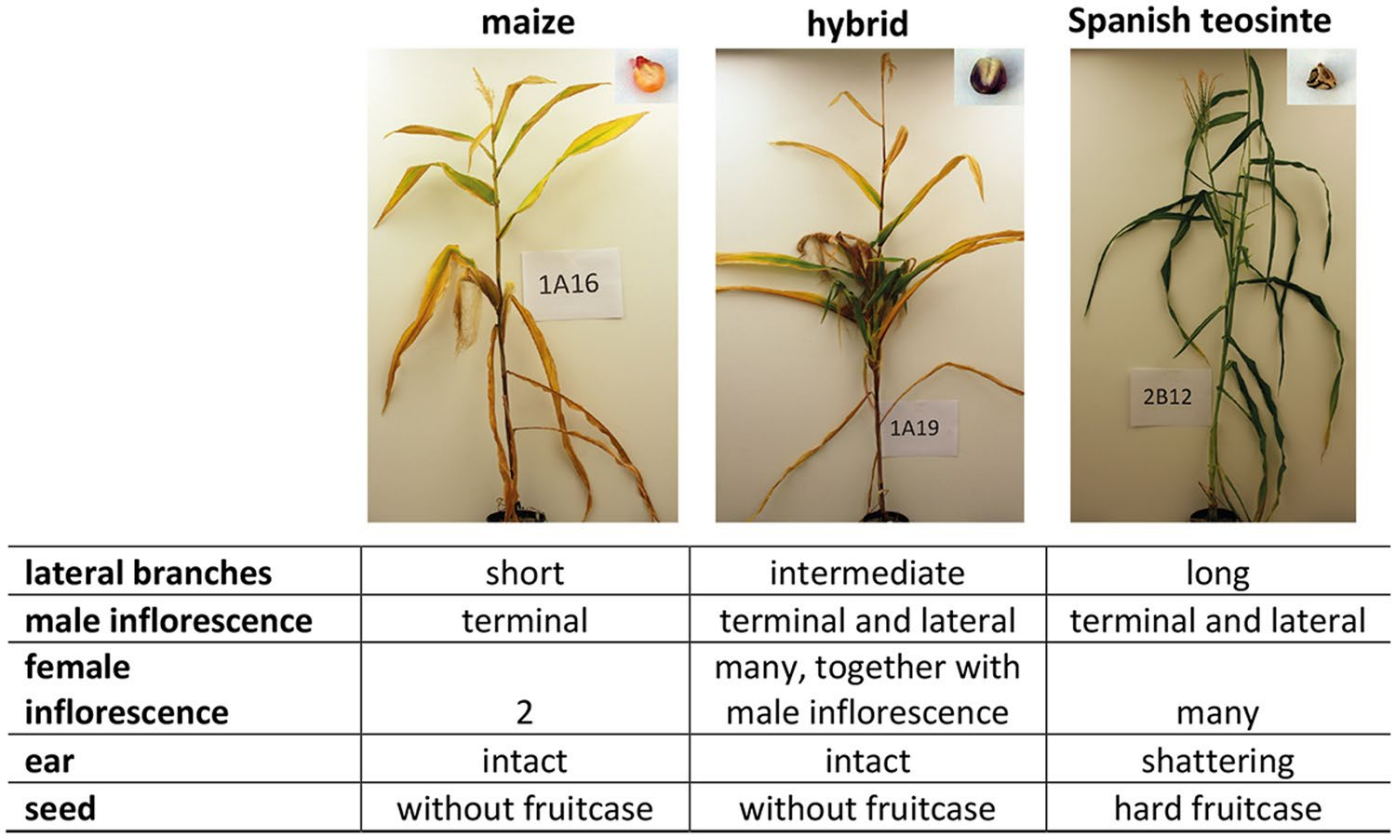
Basic morphological differences between Spanish commercial maize, hybrid and Spanish teosinte plants. Plants were grown in a climate chamber from seeds collected in the field in Spain.

Genotype data was then imported into R^39^ and individuals and loci with more than 12% and 5% missing data, respectively, were excluded from the analysis, leading to 27′476 SNPs and 662 individuals. From that dataset we used subsets of data for the different analyses. The packages adegenet^40^ and FactoMineR^41^ were used to conduct principal component analysis (PCA). Our samples from Spain were assigned into three different categories: Spanish maize, Spanish teosinte, and hybrid, based on the morphological traits shown in Fig. 5.

STRUCTURE analysis^42^ was conducted with different K’s and 10 replications using the admixture model with correlated allele frequencies. The analysis was performed separately for all Spanish maize varieties, hybrid, Spanish teosinte genotypes, *Z.m*. ssp. *mexicana and* ssp. *parviglumis* (Fig. 3) and for all commercial maize varieties, Spanish teosinte, *Z.m*. ssp. *mexicana*, ssp. *parviglumis, Z. luxurians, Z. diploperennis, Z. perennis* and *Z. nicaraguensis* (Supplementary Fig. S1). The best K was then selected using STRUCTURE harvester^43^.

To simulate the putative hybrids between the Spanish maize and *Z.m*. ssp. *mexicana* we use the hybrid function of adegenet^40^ with 10 individuals each.

## Electronic supplementary material

Supplementary Information
