# Supplementary material for: Teosinte in Europe – Searching for the Origin of a Novel Weed
Source: Sci Rep. 2017 May 8;7:1560. doi: 10.1038/s41598-017-01478-w (PMC5431553; doi:10.1038/s41598-017-01478-w)
Supplement: Supplementary file 1 — Supplementary Information [file 41598_2017_1478_MOESM1_ESM.pdf]

Supplementary Information

Teosinte in Europe – Searching for the Origin of a Novel Weed

Miluse Trtikova, Andre Lohn, Rosa Binimelis, Ignacio Chapela, Bernadette Oehen, Niklaus Zemp, Alex Widmer, Angelika Hilbeck

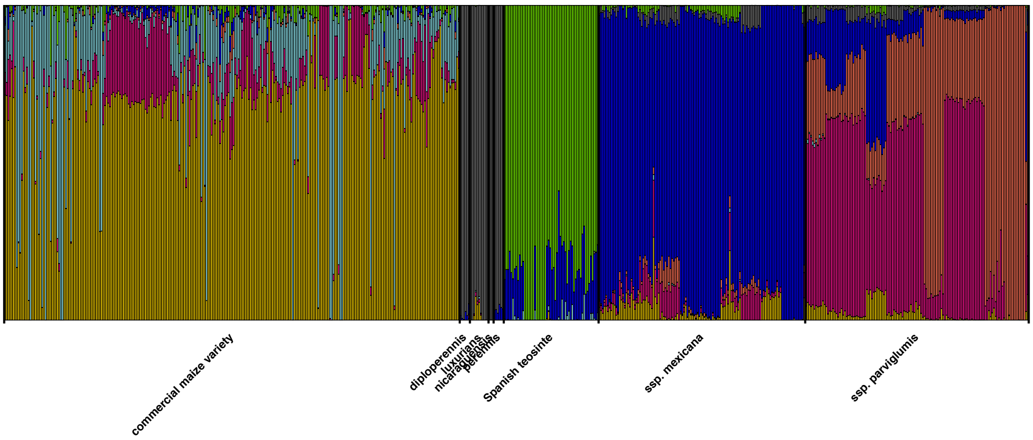

Supplementary Figure S1. Bar plot of assignment proportions from STRUCTURE analysis. At K=7 for all commercial maize varieties, Spanish teosinte, *Z.m.* ssp. *mexicana*, ssp. *parviglumis, Z. luxurians, Z. diploperennis, Z. perennis* and *Z. nicaraguensis* (27' 476 SNPs and 610 individuals).

Supplementary Table S1. List of samples used for PCA and STRUCTURE analyses. Our samples originating from Spain were combined with other publically available data.

ID	Phenotype	Origin	Variety/Accession number	From
1A1	hybrid	Spain		chamber
1A14	hybrid	Spain		chamber
1A19	hybrid	Spain		chamber
1A20	hybrid	Spain		chamber
1A23	hybrid	Spain		chamber
1A24	hybrid	Spain		chamber
1A28	hybrid	Spain		chamber
1A31	hybrid	Spain		chamber
1A31.x	hybrid	Spain		chamber

1A36	hybrid	Spain		chamber
3C1	hybrid	Spain		chamber
3C5	hybrid	Spain		chamber
3C6	hybrid	Spain		chamber
3C7	hybrid	Spain		chamber
CR1	hybrid	experimental cross	GMM x ZA5M	chamber
CR2	hybrid	experimental cross	GMM x ZA5M	chamber
CR3	hybrid	experimental cross	GMM x ZA5M	chamber
ZA4B	hybrid	Spain		chamber
ZA4D	hybrid	Spain		chamber
ZA4E	hybrid	Spain		chamber
C2	unclassified juvenile	Spain		field
C4	unclassified juvenile	Spain		field
E2	unclassified juvenile	Spain		field
E6	unclassified juvenile	Spain		field
E6.1	unclassified juvenile	Spain		field
F1_4	unclassified juvenile	Spain		field
F1_6	unclassified juvenile	Spain		field
F2_1	unclassified juvenile	Spain		field
F4	unclassified juvenile	Spain		field
E1	unclassified juvenile	Spain		field
E3	unclassified juvenile	Spain		field
EDAY2_1	unclassified juvenile	Spain		field
EDAY2_10+11	unclassified juvenile	Spain		field
EDAY2_12	unclassified juvenile	Spain		field
EDAY2_13	unclassified juvenile	Spain		field
EDAY2_14	unclassified juvenile	Spain		field
EDAY2_3	unclassified juvenile	Spain		field
EDAY2_4	unclassified juvenile	Spain		field
EDAY2_5	unclassified juvenile	Spain		field
EDAY2_6	unclassified juvenile	Spain		field
EDAY2_7	unclassified juvenile	Spain		field

EDAY2_8	unclassified juvenile	Spain		field
EDAY2_9	unclassified juvenile	Spain		field
E5	teosinte	Spain		field
A7	teosinte	Spain		field
C5	teosinte	Spain		field
C6	teosinte	Spain		field
C7	teosinte	Spain		field
C8	teosinte	Spain		field
C9	teosinte	Spain		field
D1	teosinte	Spain		field
D10	teosinte	Spain		field
D11	teosinte	Spain		field
D12	teosinte	Spain		field
D15	teosinte	Spain		field
D16	teosinte	Spain		field
D2	teosinte	Spain		field
D3	teosinte	Spain		field
D5	teosinte	Spain		field
D6	teosinte	Spain		field
D7	teosinte	Spain		field
D8	teosinte	Spain		field
D9	teosinte	Spain		field
E4	teosinte	Spain		field
E7	teosinte	Spain		field
2B12	teosinte	Spain		chamber
2B26	teosinte	Spain		chamber
2B27	teosinte	Spain		chamber
2B5	teosinte	Spain		chamber
ZA3B	teosinte	Spain		chamber
ZA3C	teosinte	Spain		chamber
ZA5B	teosinte	Spain		chamber
ZA5C	teosinte	Spain		chamber
ZA5M	teosinte	Spain		chamber
ZA6B	teosinte	Spain		chamber
ZA6C	teosinte	Spain		chamber
ZA6E	teosinte	Spain		chamber
ZA7B	teosinte	Spain		chamber
ZA7C	teosinte	Spain		chamber
ZA1A	teosinte	Spain		chamber
ZA1C	teosinte	Spain		chamber
ZA1D	teosinte	Spain		chamber
ZA1E	teosinte	Spain		chamber
1A30	maize	Spain		chamber
A1_1	maize	Spain		field

A2	maize	Spain		field
A3	maize	Spain		field
A4	maize	Spain		field
A5	maize	Spain		field
B1	maize	Spain		field
B2.x	maize	Spain		field
B3	maize	Spain		field
B4	maize	Spain		field
D14	maize	Spain		field
F3	maize	Spain		field
F5	maize	Spain		field
F6.x	maize	Spain		field
G1	maize	Spain		field
H1_1	maize	Spain		field
H2_1	maize	Spain		field
H2_2	maize	Spain		field
H3	maize	Spain		field
H4	maize	Spain		field
D13	maize	Spain		field
A6	maize	Spain		field
ISO	maize	Spain	ES TORQUAZ	chamber
OPV	maize	Spain	ROJO	chamber
GM1	maize	Spain	LG30490YG	chamber
GM2	maize	Spain	PR33D48	chamber
GMM	maize	Spain	LG30490YG	chamber
CONV	maize	Spain	DKC66-66	chamber
T14	diploperennis	USDA	PI 441930	chamber
T15	diploperennis	USDA	PI 441930	chamber
T16	diploperennis	USDA	PI 441930	chamber
T17	diploperennis	USDA	PI 462368	chamber
T18	diploperennis	USDA	PI 462368	chamber
T19	diploperennis	USDA	PI 462368	chamber
A	luxurians	CIMMYT	CIMMYTMA 30082	chamber
B	luxurians	CIMMYT	CIMMYTMA 30082	chamber
C	luxurians	CIMMYT	CIMMYTMA 30082	chamber
D	luxurians	CIMMYT	CIMMYTMA 29305	chamber
E	luxurians	CIMMYT	CIMMYTMA 29305	chamber
F	luxurians	CIMMYT	CIMMYTMA 29305	chamber
BR1	luxurians	Brazil		chamber
BR2	luxurians	Brazil		chamber
BR3	luxurians	Brazil		chamber
BR4	luxurians	Brazil		chamber
BR5	luxurians	Brazil		chamber
T3	ssp. parviglumis	USDA	AMES 21785	chamber
T4	ssp. parviglumis	USDA	AMES 21889	chamber

T10	perennis	USDA	AMES 21874	chamber
T11	perennis	USDA	AMES 21874	chamber
T12	perennis	USDA	AMES 21874	chamber
T13	perennis	USDA	AMES 21874	chamber
T8.x	perennis	USDA	AMES 21874	chamber
T9	perennis	USDA	AMES 21874	chamber
T1	ssp. mexicana	USDA	AMES 8083	chamber
T2	ssp. mexicana	USDA	PI 566674	chamber
T5	nicaraguensis	USDA	PI 615697	chamber
T6	nicaraguensis	USDA	PI 615697	chamber
T7	nicaraguensis	USDA	PI 615697	chamber
A188	maize	other		
A239	maize	other		
A272	maize	other		
A441.5	maize	other		
A554	maize	other		
A619	maize	other		
A632	maize	other		
A634	maize	other		
A635	maize	other		
A641	maize	other		
A654	maize	other		
A659	maize	other		
A661	maize	other		
A679	maize	other		
A680	maize	other		
A682	maize	other		
AB28A	maize	other		
B10	maize	other		
B103	maize	other		
B104	maize	other		
B105	maize	other		
B109	maize	other		
B115	maize	other		
B14A	maize	other		
B164	maize	other		
B2.y	maize	other		
B37	maize	other		
B46	maize	other		
B52	maize	other		
B57	maize	other		
B64	maize	other		
B68	maize	other		
B73	maize	other		
B73HTRHM	maize	other		

B75	maize	other		
B76	maize	other		
B77	maize	other		
B79	maize	other		
B84	maize	other		
B97	maize	other		
C103	maize	other		
C123	maize	other		
C49A	maize	other		
CH701.30	maize	other		
CH9	maize	other		
CI.7	maize	other		
CI187.2	maize	other		
CI21E	maize	other		
CI28A	maize	other		
CI31A	maize	other		
CI3A	maize	other		
CI64	maize	other		
CI66	maize	other		
CI90C	maize	other		
CI91B	maize	other		
CM105	maize	other		
CM174	maize	other		
CM37	maize	other		
CM7	maize	other		
CML10	maize	other		
CML103	maize	other		
CML108	maize	other		
CML11	maize	other		
CML14	maize	other		
CML154Q	maize	other		
CML157Q	maize	other		
CML158Q	maize	other		
CML218	maize	other		
CML220	maize	other		
CML228	maize	other		
CML238	maize	other		
CML247	maize	other		
CML254	maize	other		
CML258	maize	other		
CML261	maize	other		
CML264	maize	other		
CML277	maize	other		
CML281	maize	other		
CML287	maize	other		

CML311	maize	other		
CML314	maize	other		
CML321	maize	other		
CML322	maize	other		
CML323	maize	other		
CML328	maize	other		
CML331	maize	other		
CML332	maize	other		
CML333	maize	other		
CML341	maize	other		
CML38	maize	other		
CML45	maize	other		
CML5	maize	other		
CML52	maize	other		
CML61	maize	other		
CML69	maize	other		
CML77	maize	other		
CML91	maize	other		
CML92	maize	other		
CMV3	maize	other		
CO106	maize	other		
CO125	maize	other		
CO255	maize	other		
DE_2	maize	other		
DE_3	maize	other		
DE1	maize	other		
DE811	maize	other		
E2558W	maize	other		
EP1	maize	other		
F2834T	maize	other		
F44	maize	other		
F6.y	maize	other		
F7	maize	other		
GA209	maize	other		
GT112	maize	other		
H105W	maize	other		
H49	maize	other		
H84	maize	other		
H91	maize	other		
H95	maize	other		
H99	maize	other		
HI27	maize	other		
HP301	maize	other		
HY	maize	other		
I137TN	maize	other		

I205	maize	other		
I29	maize	other		
IA2132	maize	other		
IA5125	maize	other		
IDS28	maize	other		
IDS69	maize	other		
IDS91	maize	other		
IL101	maize	other		
IL14H	maize	other		
IL677A	maize	other		
K148	maize	other		
K4	maize	other		
K55	maize	other		
K64	maize	other		
KI11	maize	other		
KI14	maize	other		
KI2021	maize	other		
KI21	maize	other		
KI3	maize	other		
KI43	maize	other		
KI44	maize	other		
KY21	maize	other		
KY226	maize	other		
KY228	maize	other		
L317	maize	other		
L578	maize	other		
M14	maize	other		
M162W	maize	other		
M37W	maize	other		
MEF156.55.2	maize	other		
MO17	maize	other		
MO18W	maize	other		
MO1W	maize	other		
MO24W	maize	other		
MO44	maize	other		
MO45	maize	other		
MO46	maize	other		
MO47	maize	other		
MOG	maize	other		
MP339	maize	other		
MS1334	maize	other		
MS153	maize	other		
MS71	maize	other		
MT42	maize	other		
N192	maize	other		

N28HT	maize	other		
N6	maize	other		
N7A	maize	other		
NC222	maize	other		
NC230	maize	other		
NC232	maize	other		
NC236	maize	other		
NC238	maize	other		
NC250	maize	other		
NC258	maize	other		
NC260	maize	other		
NC262	maize	other		
NC264	maize	other		
NC290A	maize	other		
NC294	maize	other		
NC296	maize	other		
NC296A	maize	other		
NC298	maize	other		
NC300	maize	other		
NC302	maize	other		
NC304	maize	other		
NC306	maize	other		
NC310	maize	other		
NC314	maize	other		
NC318	maize	other		
NC320	maize	other		
NC324	maize	other		
NC326	maize	other		
NC328	maize	other		
NC33	maize	other		
NC336	maize	other		
NC340	maize	other		
NC342	maize	other		
NC344	maize	other		
NC346	maize	other		
NC348	maize	other		
NC350	maize	other		
NC352	maize	other		
NC354	maize	other		
NC356	maize	other		
NC358	maize	other		
NC360	maize	other		
NC362	maize	other		
NC364	maize	other		
NC366	maize	other		

NC368	maize	other		
ND246	maize	other		
OH40B	maize	other		
OH43	maize	other		
OH43E	maize	other		
OH603	maize	other		
OH7B	maize	other		
OS420	maize	other		
P39	maize	other		
PA762	maize	other		
PA875	maize	other		
PA880	maize	other		
PA91	maize	other		
R177	maize	other		
R229	maize	other		
R4	maize	other		
SA24	maize	other		
SC213R	maize	other		
SC357	maize	other		
SC55	maize	other		
SD40	maize	other		
SD44	maize	other		
SG1533	maize	other		
SG18	maize	other		
T232	maize	other		
T234	maize	other		
T8.y	maize	other		
TX303	maize	other		
TX601	maize	other		
TZI10	maize	other		
TZI11	maize	other		
TZI16	maize	other		
TZI18	maize	other		
TZI25	maize	other		
TZI9	maize	other		
U267Y	maize	other		
VA102	maize	other		
VA14	maize	other		
VA17	maize	other		
VA22	maize	other		
VA35	maize	other		
VA59	maize	other		
VA85	maize	other		
VA99	maize	other		
VAW6	maize	other		

W117HT	maize	other		
W153R	maize	other		
W182B	maize	other		
W22	maize	other		
W22_R	maize	other		
WD	maize	other		
WF9	maize	other		
X33.16	maize	other		
X38.11	maize	other		
X4226	maize	other		
X4722	maize	other		
YU796	maize	other		
mex_RIMME0021.C1.1.1	ssp. mexicana	other		
mex_RIMME0021.C10.1.1	ssp. mexicana	other		
mex_RIMME0021.C11.1.1	ssp. mexicana	other		
mex_RIMME0021.C12.1.1	ssp. mexicana	other		
mex_RIMME0021.C13.1.1	ssp. mexicana	other		
mex_RIMME0021.C14.1.1	ssp. mexicana	other		
mex_RIMME0021.C2.1.1	ssp. mexicana	other		
mex_RIMME0021.C3.1.1	ssp. mexicana	other		
mex_RIMME0021.C4.1.1	ssp. mexicana	other		
mex_RIMME0021.C6.1.1	ssp. mexicana	other		
mex_RIMME0021.C7.1.1	ssp. mexicana	other		
mex_RIMME0021.C9.1.1	ssp. mexicana	other		
mex_RIMME0026.C10.1.1	ssp. mexicana	other		
mex_RIMME0026.C11.1.1	ssp. mexicana	other		
mex_RIMME0026.C12.1.1	ssp. mexicana	other		
mex_RIMME0026.C14.1.1	ssp. mexicana	other		
mex_RIMME0026.C15.1.1	ssp. mexicana	other		
mex_RIMME0026.C3.1.1	ssp. mexicana	other		
mex_RIMME0026.C4.1.1	ssp. mexicana	other		
mex_RIMME0026.C5.1.1	ssp. mexicana	other		
mex_RIMME0026.C6.1.1	ssp. mexicana	other		
mex_RIMME0026.C7.1.1	ssp. mexicana	other		
mex_RIMME0026.C8.1.1	ssp. mexicana	other		
mex_RIMME0026.C9.1.1	ssp. mexicana	other		
mex_RIMME0028.1	ssp. mexicana	other		
mex_RIMME0028.10	ssp. mexicana	other		
mex_RIMME0028.13	ssp. mexicana	other		
mex_RIMME0028.14	ssp. mexicana	other		
mex_RIMME0028.2	ssp. mexicana	other		
mex_RIMME0028.3	ssp. mexicana	other		
mex_RIMME0028.4	ssp. mexicana	other		
mex_RIMME0028.5	ssp. mexicana	other		
mex_RIMME0028.6	ssp. mexicana	other		

mex_RIMME0028.7	ssp. mexicana	other		
mex_RIMME0028.8	ssp. mexicana	other		
mex_RIMME0028.9	ssp. mexicana	other		
mex_RIMME0029.1	ssp. mexicana	other		
mex_RIMME0029.10	ssp. mexicana	other		
mex_RIMME0029.11	ssp. mexicana	other		
mex_RIMME0029.12	ssp. mexicana	other		
mex_RIMME0029.13	ssp. mexicana	other		
mex_RIMME0029.14	ssp. mexicana	other		
mex_RIMME0029.2	ssp. mexicana	other		
mex_RIMME0029.3	ssp. mexicana	other		
mex_RIMME0029.4	ssp. mexicana	other		
mex_RIMME0029.6	ssp. mexicana	other		
mex_RIMME0029.8	ssp. mexicana	other		
mex_RIMME0029.9	ssp. mexicana	other		
mex_RIMME0030.1	ssp. mexicana	other		
mex_RIMME0030.11	ssp. mexicana	other		
mex_RIMME0030.12	ssp. mexicana	other		
mex_RIMME0030.13	ssp. mexicana	other		
mex_RIMME0030.14	ssp. mexicana	other		
mex_RIMME0030.2	ssp. mexicana	other		
mex_RIMME0030.4	ssp. mexicana	other		
mex_RIMME0030.5	ssp. mexicana	other		
mex_RIMME0030.6	ssp. mexicana	other		
mex_RIMME0030.7	ssp. mexicana	other		
mex_RIMME0030.8	ssp. mexicana	other		
mex_RIMME0030.9	ssp. mexicana	other		
mex_RIMME0031.1	ssp. mexicana	other		
mex_RIMME0031.10	ssp. mexicana	other		
mex_RIMME0031.11	ssp. mexicana	other		
mex_RIMME0031.12	ssp. mexicana	other		
mex_RIMME0031.2	ssp. mexicana	other		
mex_RIMME0031.3	ssp. mexicana	other		
mex_RIMME0031.4	ssp. mexicana	other		
mex_RIMME0031.5	ssp. mexicana	other		
mex_RIMME0031.6	ssp. mexicana	other		
mex_RIMME0031.7	ssp. mexicana	other		
mex_RIMME0031.8	ssp. mexicana	other		
mex_RIMME0031.9	ssp. mexicana	other		
mex_RIMME0032.1	ssp. mexicana	other		
mex_RIMME0032.10	ssp. mexicana	other		
mex_RIMME0032.12	ssp. mexicana	other		
mex_RIMME0032.13	ssp. mexicana	other		
mex_RIMME0032.14	ssp. mexicana	other		
mex_RIMME0032.15	ssp. mexicana	other		

mex_RIMME0032.3	ssp. mexicana	other		
mex_RIMME0032.4	ssp. mexicana	other		
mex_RIMME0032.5	ssp. mexicana	other		
mex_RIMME0032.7	ssp. mexicana	other		
mex_RIMME0032.8	ssp. mexicana	other		
mex_RIMME0032.9	ssp. mexicana	other		
mex_RIMME0033.1	ssp. mexicana	other		
mex_RIMME0033.10	ssp. mexicana	other		
mex_RIMME0033.11	ssp. mexicana	other		
mex_RIMME0033.12	ssp. mexicana	other		
mex_RIMME0033.13	ssp. mexicana	other		
mex_RIMME0033.3_dup1	ssp. mexicana	other		
mex_RIMME0033.4	ssp. mexicana	other		
mex_RIMME0033.5	ssp. mexicana	other		
mex_RIMME0033.6	ssp. mexicana	other		
mex_RIMME0033.7	ssp. mexicana	other		
mex_RIMME0033.8	ssp. mexicana	other		
mex_RIMME0033.9	ssp. mexicana	other		
mex_RIMME0034.1	ssp. mexicana	other		
mex_RIMME0034.10	ssp. mexicana	other		
mex_RIMME0034.12	ssp. mexicana	other		
mex_RIMME0034.13	ssp. mexicana	other		
mex_RIMME0034.2	ssp. mexicana	other		
mex_RIMME0034.3	ssp. mexicana	other		
mex_RIMME0034.4	ssp. mexicana	other		
mex_RIMME0034.5	ssp. mexicana	other		
mex_RIMME0034.6	ssp. mexicana	other		
mex_RIMME0034.7	ssp. mexicana	other		
mex_RIMME0034.8	ssp. mexicana	other		
mex_RIMME0034.9	ssp. mexicana	other		
mex_RIMME0035.1	ssp. mexicana	other		
mex_RIMME0035.10	ssp. mexicana	other		
mex_RIMME0035.11	ssp. mexicana	other		
mex_RIMME0035.12	ssp. mexicana	other		
mex_RIMME0035.2	ssp. mexicana	other		
mex_RIMME0035.3	ssp. mexicana	other		
mex_RIMME0035.4	ssp. mexicana	other		
mex_RIMME0035.5	ssp. mexicana	other		
mex_RIMME0035.6	ssp. mexicana	other		
mex_RIMME0035.7	ssp. mexicana	other		
mex_RIMME0035.8	ssp. mexicana	other		
mex_RIMME0035.9	ssp. mexicana	other		
par_RIMPA0071.1	ssp. parviglumis	other		
par_RIMPA0071.10	ssp. parviglumis	other		
par_RIMPA0071.11	ssp. parviglumis	other		

par_RIMPA0071.12	ssp. parviglumis	other		
par_RIMPA0071.13	ssp. parviglumis	other		
par_RIMPA0071.14	ssp. parviglumis	other		
par_RIMPA0071.16	ssp. parviglumis	other		
par_RIMPA0071.17	ssp. parviglumis	other		
par_RIMPA0071.2	ssp. parviglumis	other		
par_RIMPA0071.3	ssp. parviglumis	other		
par_RIMPA0071.4	ssp. parviglumis	other		
par_RIMPA0071.5	ssp. parviglumis	other		
par_RIMPA0086.01	ssp. parviglumis	other		
par_RIMPA0086.02	ssp. parviglumis	other		
par_RIMPA0086.03	ssp. parviglumis	other		
par_RIMPA0086.04	ssp. parviglumis	other		
par_RIMPA0086.06	ssp. parviglumis	other		
par_RIMPA0086.07	ssp. parviglumis	other		
par_RIMPA0086.08	ssp. parviglumis	other		
par_RIMPA0086.09	ssp. parviglumis	other		
par_RIMPA0086.10	ssp. parviglumis	other		
par_RIMPA0086.11	ssp. parviglumis	other		
par_RIMPA0086.12	ssp. parviglumis	other		
par_RIMPA0086.13	ssp. parviglumis	other		
par_RIMPA0087.01	ssp. parviglumis	other		
par_RIMPA0087.02	ssp. parviglumis	other		
par_RIMPA0087.03	ssp. parviglumis	other		
par_RIMPA0087.06	ssp. parviglumis	other		
par_RIMPA0087.07	ssp. parviglumis	other		
par_RIMPA0087.08	ssp. parviglumis	other		
par_RIMPA0087.09	ssp. parviglumis	other		
par_RIMPA0087.10	ssp. parviglumis	other		
par_RIMPA0087.11	ssp. parviglumis	other		
par_RIMPA0087.12	ssp. parviglumis	other		
par_RIMPA0087.4	ssp. parviglumis	other		
par_RIMPA0087.5	ssp. parviglumis	other		
par_RIMPA0096.01	ssp. parviglumis	other		
par_RIMPA0096.02	ssp. parviglumis	other		
par_RIMPA0096.03	ssp. parviglumis	other		
par_RIMPA0096.11	ssp. parviglumis	other		
par_RIMPA0096.12	ssp. parviglumis	other		
par_RIMPA0096.13	ssp. parviglumis	other		
par_RIMPA0096.14	ssp. parviglumis	other		
par_RIMPA0096.15	ssp. parviglumis	other		
par_RIMPA0096.17	ssp. parviglumis	other		
par_RIMPA0096.6	ssp. parviglumis	other		
par_RIMPA0096.7	ssp. parviglumis	other		
par_RIMPA0096.8	ssp. parviglumis	other		

par_RIMPA0110.10	ssp. parviglumis	other		
par_RIMPA0110.11	ssp. parviglumis	other		
par_RIMPA0110.2_dup1	ssp. parviglumis	other		
par_RIMPA0110.3_dup2	ssp. parviglumis	other		
par_RIMPA0110.4_dup1	ssp. parviglumis	other		
par_RIMPA0110.5	ssp. parviglumis	other		
par_RIMPA0110.6	ssp. parviglumis	other		
par_RIMPA0110.7	ssp. parviglumis	other		
par_RIMPA0110.8	ssp. parviglumis	other		
par_RIMPA0110.9	ssp. parviglumis	other		
par_RIMPA0135.01	ssp. parviglumis	other		
par_RIMPA0135.02	ssp. parviglumis	other		
par_RIMPA0135.03	ssp. parviglumis	other		
par_RIMPA0135.04	ssp. parviglumis	other		
par_RIMPA0135.05	ssp. parviglumis	other		
par_RIMPA0135.07	ssp. parviglumis	other		
par_RIMPA0135.08_dup1	ssp. parviglumis	other		
par_RIMPA0135.09	ssp. parviglumis	other		
par_RIMPA0135.10	ssp. parviglumis	other		
par_RIMPA0135.11	ssp. parviglumis	other		
par_RIMPA0135.12	ssp. parviglumis	other		
par_RIMPA0135.13	ssp. parviglumis	other		
par_RIMPA0142.01	ssp. parviglumis	other		
par_RIMPA0142.03	ssp. parviglumis	other		
par_RIMPA0142.04	ssp. parviglumis	other		
par_RIMPA0142.06	ssp. parviglumis	other		
par_RIMPA0142.07	ssp. parviglumis	other		
par_RIMPA0142.08	ssp. parviglumis	other		
par_RIMPA0142.09	ssp. parviglumis	other		
par_RIMPA0142.11	ssp. parviglumis	other		
par_RIMPA0142.12	ssp. parviglumis	other		
par_RIMPA0142.13	ssp. parviglumis	other		
par_RIMPA0142.14	ssp. parviglumis	other		
par_RIMPA0142.2	ssp. parviglumis	other		
par_RIMPA0155.C12.1	ssp. parviglumis	other		
par_RIMPA0155.C13.1	ssp. parviglumis	other		
par_RIMPA0155.C14.1	ssp. parviglumis	other		
par_RIMPA0155.C19.1	ssp. parviglumis	other		
par_RIMPA0155.C23.1	ssp. parviglumis	other		
par_RIMPA0155.C28.1	ssp. parviglumis	other		
par_RIMPA0155.C32.1	ssp. parviglumis	other		
par_RIMPA0155.C33.1	ssp. parviglumis	other		
par_RIMPA0155.C36.1	ssp. parviglumis	other		
par_RIMPA0155.C39.1	ssp. parviglumis	other		
par_RIMPA0155.C42.1	ssp. parviglumis	other		

par_RIMPA0155.C45.1	ssp. parviglumis	other		
par_RIMPA0156C15	ssp. parviglumis	other		
par_RIMPA0156C18	ssp. parviglumis	other		
par_RIMPA0156C19	ssp. parviglumis	other		
par_RIMPA0156C21	ssp. parviglumis	other		
par_RIMPA0156C28	ssp. parviglumis	other		
par_RIMPA0156C29	ssp. parviglumis	other		
par_RIMPA0156C38	ssp. parviglumis	other		
par_RIMPA0156C42	ssp. parviglumis	other		
par_RIMPA0156C43	ssp. parviglumis	other		
par_RIMPA0156C45	ssp. parviglumis	other		
par_RIMPA0156C48	ssp. parviglumis	other		
par_RIMPA0156C49	ssp. parviglumis	other		
par_RIMPA0157.C10.1	ssp. parviglumis	other		
par_RIMPA0157.C15.1	ssp. parviglumis	other		
par_RIMPA0157.C16.1	ssp. parviglumis	other		
par_RIMPA0157.C19.1	ssp. parviglumis	other		
par_RIMPA0157.C20.1	ssp. parviglumis	other		
par_RIMPA0157.C22.1	ssp. parviglumis	other		
par_RIMPA0157.C3.1	ssp. parviglumis	other		
par_RIMPA0157.C32.1	ssp. parviglumis	other		
par_RIMPA0157.C39.1	ssp. parviglumis	other		
par_RIMPA0157.C45.1	ssp. parviglumis	other		
par_RIMPA0157.C48.1	ssp. parviglumis	other		
par_RIMPA0157.C9.1	ssp. parviglumis	other		
par_RIMPA0158C13	ssp. parviglumis	other		
par_RIMPA0158C15	ssp. parviglumis	other		
par_RIMPA0158C21	ssp. parviglumis	other		
par_RIMPA0158C27	ssp. parviglumis	other		
par_RIMPA0158C29	ssp. parviglumis	other		
par_RIMPA0158C3	ssp. parviglumis	other		
par_RIMPA0158C33	ssp. parviglumis	other		
par_RIMPA0158C44	ssp. parviglumis	other		
par_RIMPA0158C46	ssp. parviglumis	other		
par_RIMPA0158C49	ssp. parviglumis	other		
par_RIMPA0158C6	ssp. parviglumis	other		
par_RIMPA158.C50..1	ssp. parviglumis	other		
